# Exosome-mediated human norovirus infection

**DOI:** 10.1371/journal.pone.0237044

**Published:** 2020-08-03

**Authors:** Kyle V. Todd, Ralph A. Tripp

**Affiliations:** Department of Infectious Diseases, University of Georgia, Athens, GA, United States of America; University of Illinois at Chicago College of Medicine, UNITED STATES

## Abstract

Human norovirus (HuNoV) is a leading cause of acute gastroenteritis. Outbreaks normally occur via the fecal-oral route. HuNoV infection is thought to occur by viral particle transmission, but increasing evidence suggests a function for exosomes in HuNoV infection. HuNoV is contained within stool-derived exosomes, and exosome-associated HuNoV has been shown to replicate in human intestinal enteroids. In this study, we examine exosome-associated HuNoV infection of Vero cells and show that exosomes containing HuNoV may attach, infect, and be passaged in Vero cells. These findings support earlier findings and have implications for developing HuNoV disease intervention strategies.

## Introduction

Noroviruses (NoVs) are non-enveloped enteric viruses with small capsids that contain positive-sense RNA genomes [1, 2]. NoVs are classified into ten genogroups (GI-GX), with GI, GII, and GIV typically causing the majority of human disease [3]. Human NoVs (HuNoVs) are globally prevalent and often cause self-limiting acute gastroenteritis. There are ~700 million infections and >200,000 deaths that occur from HuNoV infections annually [4]. GII genotype 4 (GII.4) HuNoVs are a concern because they may cause pandemics giving rise to dominant circulating strains [5–7].

There remains a need for HuNoV disease interventions including vaccines to prevent disease. Human intestinal enteroids (HIEs) [8] and human Burkitt lymphoma B cells (BJAB cells) [9] have produced encouraging results as HuNoV cell culture models, however, these substrates are not ideal for vaccine production because HIEs are terminal mixed cell culture systems with a limited lifespan [8, 10] and BJAB cells have been reported to support only a single strain of HuNoV [9, 11]. A recent study from our group has shown that Vero cells support HuNoV infection and replication [12], and as Vero cells are used for the production of a variety of viral vaccines [13], the study was also encouraging for potential HuNoV vaccine production.

Infection and transmission of HuNoV are considered to occur by viral particles, however, accumulating evidence suggests that HuNoVs co-opt host cell endosome pathways for transmission [14–16]. Briefly, endocytosis of extracellular components generates endosomes in mammalian cells. These vesicles recycle and fuse leading to the formation of multivesicular bodies (MVBs). MVBs contain intraluminal vesicles that form by vesicle fusion and invagination of the endosomal membrane [17, 18]. These intraluminal vesicles and their cargo are either degraded following fusion of MVBs with lysosomes, or released extracellularly as single-membraned vesicles called exosomes [19]. Exosomes are membrane-bound extracellular vesicles (EVs) ranging in size from 50–150 nm. Exosomes may carry cell-specific cargo loads of proteins, lipids, and/or nucleic acids that may be selectively taken up by neighboring or distant cells [20, 21]. Exosome loading is regulated in part by post-translational protein modifications including ISGylation, phosphorylation, SUMOylation, and ubiquitination [20, 22–24]. Several non-enveloped viruses, e.g. hepatitis A virus [25], hepatitis E virus [26–28], poliovirus [29], and rotavirus [14] can transmit to other cells using EVs. Transport within EVs allows viruses to remain immunologically undetected, and to cluster enhancing the likelihood of infection. Exosomes can regulate virus infections as they may shuttle microRNAs and cytokines to neighboring cells inducing an antiviral state [30, 31]. Exosomes are stable in body fluids, including stool [12, 14, 32]. HuNoV RNA is also contained within exosomes, and these exosomes are infectious in HIEs [12, 14]. Given the importance of exosomes for HuNoV infection and transmission, we examined the potential of HuNoV stool-derived exosomes to internalize, replicate, and be passaged in Vero cells.

## Materials and methods

### Cells

Vero cells (African green monkey kidney cells; CCL81.4) were obtained from the American Type Culture Collection (ATCC; Manassas, VA) and maintained at low passage (passages 25–30) in Dulbecco’s Modified Eagle’s Media (DMEM; GIBCO, Gaithersburg, MD), with 5% heat-inactivated fetal bovine serum (FBS; HyClone, Logan, UT), at 37°C and 5% CO_2_. A master cell bank was generated to ensure low passage cells were used for all experiments.

### Viruses

Deidentified human stool samples containing GII.3, GII.4 Den Haag (2006b pandemic), or GII.4 Sydney (2012 pandemic) HuNoV were acquired from Murdoch Children’s Research Institute (MCRI; Melbourne Australia) or the Viral Gastroenteritis Branch in the Division of Viral Diseases in the Centers for Disease Control and Prevention (CDC, Atlanta, GA) and stored at -80°C. The stool samples were thawed on ice before making 10% (w/v) dilutions in PBS (HyClone). All samples were centrifuged 2x at 1500xg for 10 min at 4°C and then at 5000xg for 10 min at 4°C. The stool samples were passed through 100 μm and 40 μm cell strainers (BD, Franklin Lakes, NJ) then filtered samples were filtered using 0.45 μm filters (GE Healthcare, Chicago, IL) before aliquots were made and stored at -80°C until use.

### HuNoV genome equivalent (g.e.) quantification

Stool samples were treated with RNAzol (Molecular Research Center Inc., Cincinnati, OH) according to the manufacturer’s instructions to generate total RNA to be used for amplification and detection by qRT-PCR [12]. Briefly, NK2P_2_F (+) and NKP_2_R (−) primers were used to amplify a segment of the HuNoV RNA-dependent RNA polymerase (RdRp) that was detected using the RING_2_-TP probe under the following cycling conditions: 45°C for 10 min, 95°C for 10 min, followed by 40 cycles of 95°C for 15 sec, 50°C for 30 sec, and 60°C for 30 sec. This HuNoV qRT-PCR procedure has excellent sensitivity (96.1%) and specificity (97.7%) [33]. Here we report an average intra-assay coefficient of variation for the qRT-PCR standards of 0.63% ± 0.07 standard error of the mean (SEM). The resulting RdRp qRT-PCR levels are considered g.e. because the amplified site occurs once in each full-length genome. The g.e. RNA levels for the experimental time-points were divided by the mean g.e. RNA levels from the input time-point (i.e. 0h) to calculate fold-changes, normalizing the fold-change of input time-points to 1. MOI was calculated as the ratio of input g.e. to the number of cells.

### Exosome isolation

The stool was processed with ExoQuick (System Biosciences, Palo Alto, CA) to isolate exosomes according to the manufacturer’s instructions. Briefly, stool samples were centrifuged at 3000xg for 15 min, then supernatants were mixed with ExoQuick (final concentration 20%) before refrigeration at 4°C for 16h. The mixture was subsequently centrifuged at 1500xg for 30 min and the supernatant was removed. Exosome pellets were resuspended in PBS and stored at -80°C until use. Stool exosome samples were tested 10x using a NS300 NanoSight (Spectris, Egham, GBR) for the determination of EV concentrations and sizes (S1 Fig).

### Phosphatidylserine (PS) exosome partitioning

Stool samples were processed using a MagCapture Exosome Isolation kit PS (FUJIFILM Wako Chemicals, Richmond, VA) to enrich for PS-exosomes. Briefly, a supplied exosome capture immobilizing buffer was added to streptavidin magnetic beads, mixed, then placed on a magnetic stand before removal of the supernatant. Exosome capture immobilizing buffer and biotin-labeled exosome buffer were mixed with the streptavidin magnetic beads and rotated at 4°C for 10 min. After centrifugation at 1000xg for 1 min, the beads were placed on a magnetic stand and the supernatant was removed. Subsequently, the streptavidin magnetic beads were washed 2x with exosome capture immobilizing buffer. An exosome binding enhancer buffer was diluted 1:500 in the stool before incubation with the streptavidin magnetic beads at 4°C for 3h. Following centrifugation at 1000xg for 1 min, and placement on a magnetic stand, the PS-exosome-depleted stool was collected and stored at -80°C until use. PS-exosome bound streptavidin magnetic beads were washed 2x with washing buffer containing 1:500 diluted exosome binding enhancer. The PS-exosomes were eluted 2x with the exosome elution buffer then stored at -80°C until use.

### HuNoV infection and passage in Vero cells

Stool, exosomes, PS-exosomes, or PS-exosome depleted stool was used to infect (MOIs described in figure legends) Vero cells. Briefly, 8.0x10^3^ Vero cells were plated in 96-well flat-bottom plates (Corning, Corning, NY), the supernatant decanted, and serum free (SF)-DMEM volume-normalized (10 μL) HuNoV infections were performed in 90 μL of SF-DMEM for the duration of the experimental time-course. All HuNoV infections were performed in SF-DMEM to avoid the presence of cofounding exosomes from FBS [34]. The cells were gently rocked then incubated at 37°C and 5% CO_2_. For 1h and 6h infections (Table 1), the infectious supernatants were removed from all wells except virus input controls, and the cells were washed 2x with PBS. RNAzol was used to extract RNA from the cellular fractions of experimental wells and from the cells and supernatants of virus input controls. The % total g.e. was determined by dividing the cellular fraction g.e. by the virus input controls across triplicate experimental and control wells. For exosome passage studies, 10^6^ Vero cells were plated in T-25 flasks (Corning), the media decanted, and SF-DMEM volume-normalized (100 μL) HuNoV infections (MOI = 0.5) were performed in SF-DMEM (4.9 mL). Exosomes were isolated from 5 mL of infectious supernatants from each flask 72hpi using ExoQuick, then the g.e. of the exosomes was quantified by qRT-PCR. Isolated exosomes were volume-normalized in SF-DMEM (10 μL) before infection (MOI = 0.2) of 8.0x10^3^ fresh Vero cells in 90 μL of SF-DMEM in 96-well flat-bottom plates for 72h.

**Table 1:** GII.4 Sydney HuNoV levels by qRT-PCR.

Virus	Sample	Time-point	Mean ± SEM	Significance
GII.4 Sydney	exosomes	1h	2.16 ± 0.27	–
		6h	3.96 ± 0.41	**

Percent total HuNoV g.e. displayed. Percent total HuNoV g.e. interaction with Vero cells after HuNoV infection (MOI = 1.0) is increased at 6h post-infection (hpi) compared to 1hpi. Significant differences were observed between 1h and 6h time-points. Data represent n = 3 ± SEM.

### Statistical analyses

Unpaired two-tailed t-tests were performed with 95% confidence intervals using GraphPad Prism. p-values <0.05 were considered significant: *p<0.05, **p<0.01, and ****p<0.0001. Means ± SEM are displayed.

## Results

We tested if exosomes from stool samples could infect Vero cells following incubation for 1h or 6h. The data showed that increased incubation times correlated with increased intracellular HuNoV as measured by qRT-PCR (Table 1). This observation is consistent with increased incubation times facilitating HuNoV replication in Vero cells [12]. The HuNoV g.e. levels in Vero cells were similar between stool [12] and exosome infections at both 1h and 6h time-points. PS-exosomes enriched from GII.4 Syndey or GII.3 stool transferred infectious virus that replicated in Vero cells as indicated by a significant increase in g.e. (Table 2). The depletion of PS-exosomes from GII.4 Sydney or GII.3 stool by magnetic bead partitioning did not ablate HuNoV replication in Vero cells (Table 2). This finding suggests that non-PS-exosomes contain HuNoV, and/or virus particles not in exosomes are infectious. GII.4 Den Haag stool or PS-exosomes from GII.4 Den Haag stool did not infect Vero cells (Table 2), possibly indicating strain differences for infection and replication, which is consistent with a previous attempt to infect Vero cells with a GII.4 Den Haag strain [12].

**Table 2:** Vero cells infected with PS-exosomes or PS-exosome depleted stool.

Virus	Sample	Time-point	Mean ± SEM	Significance
GII.4 Sydney	PS-exosomes	0h	1.00 ± 0.12	–
		72h	1.43 ± 0.12	*
	PS-depleted	0h	1.00 ± 0.09	–
		72h	1.94 ± 0.13	****
GII.3	PS-exosomes	0h	1.00 ± 0.08	–
		72h	1.42 ± 0.17	*
	PS-depleted	0h	1.00 ± 0.09	–
		72h	2.67 ± 0.28	****
GII.4 Den Haag	stool	0h	1.01 ± 0.13	–
		72h	1.28 ± 0.17	ns
	PS-exosomes	0h	1.00 ± 0.14	–
		72h	1.22 ± 0.18	ns
	PS-depleted	0h	0.96 ± 0.07	–
		72h	0.99 ± 0.13	ns

qRT-PCR analysis after a 72h infection (MOI = 0.1). Mean fold-changes over 0h time-points displayed. Significant differences between 0h and 72h time-points were calculated. Magnetic bead partitioning was used to isolate PS-exosomes and generate PS-depleted stool samples. Data represent n = 3 ± SEM.

In this report, we show that exosomes will transfer HuNoV infection to uninfected Vero cells (Table 3). This is the first account of HuNoV passage by exosomes, although additional passaging needs to be performed to confirm exosomes as a mode of transmission. We propose that infectious virus is loaded as cargo into exosomes and this occurs modestly but reproducibly.

**Table 3:** Infected Vero cells release HuNoV-loaded exosomes.

Virus	Sample	Time-point	Mean ± SEM	Significance
GII.4 Sydney	stool	0h	1.00 ± 0.06	–
		72h	1.44 ± 0.11	**
	exosomes	0h	1.00 ± 0.03	–
		72h	1.45 ± 0.06	****

After a 72h infection (MOI = 0.5) with either stool or stool exosomes, exosomes were isolated by ExoQuick and used to infect (MOI = 0.2) fresh Vero cells for 72h. Exosome passage was performed at a lower MOI as there was lower virus recovery after infection and exosome isolation. Significant differences between 0h and 72h time-points were calculated. Data for passage 2 are shown and represent n = 3 ± SEM.

## Discussion

Exosomes from HuNoV-containing stool samples transferred infectious HuNoV to uninfected Vero cells (Table 1) at a level similar to that previously reported for whole stool [12]. HuNoV (<1%–10%) [8, 35] and exosomes from stool (6%) [14] produced similar infections in HIEs. Exosomes from murine NoV-infected RAW264.7 macrophages have been tested and cannot infect RAW264.7 cells when the murine NoV receptor, CD300ld/CD300lf, is blocked indicating that passive exosome uptake of the virus is insufficient or not occurring [14, 36, 37]. It is unclear if the attachment and entry mechanisms of HuNoV virions and HuNoV-loaded exosomes are identical. GII.4 Sydney replication in Vero cells was low for PS-exosomes (~1.5-fold) compared to total stool exosomes (~2.5-fold) [12], or PS-exosome-depleted stool (~2-fold). Increased HuNoV replication from total stool exosomes over PS-exosomes indicates that non-PS-exosome subpopulations may also function to facilitate HuNoV infection. HuNoV in tetraspanin-containing (CD9, CD63, and CD81) exosomes has been reported [14]. Tetraspanins represent a diverse family of membrane-bound proteins that form tetraspanin-enriched microdomains, which coordinate diverse exosome processes including biogenesis, cargo loading, and recipient cell attachment (as reviewed in [38]). Their role in exosome development suggests tetraspanin-containing exosomes are notable subpopulations to study for exosome-mediated HuNoV infection. Additionally, differences between HuNoV stool and exosome replication in Vero cells may be due to the absence of specific stool components (host, viral, or bacterial) following exosome isolation, however this has not been further explored.

Previous attempts to mediate HuNoV infection in Vero cells using transfection reagents to load stool exosomes were unsuccessful [12]. This suggests that non-specific liposome encapsulation of HuNoV does not aid HuNoV infection or replication in Vero cells hinting at exosome loading specificity. Additionally, exosomes from uninfected Vero cells or Caco-2 cells (an intestinal epithelial cell line) co-administered with HuNoV (MOI = 1.0) had no detectable effect on HuNoV replication.

It is appreciated that non-enveloped viruses are released after inducing host cell lysis. Interestingly, HuNoV replication within BJAB cells [9], HIEs [8], and Vero cells [12] do not cause cell lysis. This indicates that the replication of HuNoV in these cell culture models is likely the result of non-lytic spread. It has been reported that the majority of murine NoV is released before cell membrane lysis [14], meaning that the non-lytic spread of NoVs may occur more readily than previously thought. The upregulation of pathways associated with non-lytic spread by bile acid supplementation [15] enhances HuNoV infection in HIEs [8]. Mounting *in vitro* evidence supports the non-lytic spread of HuNoVs, however, *in vivo* experiments are needed to further validate its role in infection and transmission.

## Supporting information

S1 Fig ExoQuick-isolated exosomes. NanoSight analysis indicated that high concentrations of exosomes (50–150 nm) are contained within HuNoV-infected stool. Extracellular vesicle (EV).
(DOCX)

S2 Fig Sample HuNoV g.e. standard curve. (DOCX)

S1 Table Mean Ct values associated with Table 2. (DOCX)

S2 Table Mean Ct values associated with Table 3. (DOCX)
