# Supplementary material for: Exosome-mediated human norovirus infection
Source: PLoS One. 2020 Aug 3;15(8):e0237044. doi: 10.1371/journal.pone.0237044 (PMC7398508; doi:10.1371/journal.pone.0237044)

**S1 Fig. ExoQuick-isolated exosomes.** NanoSight analysis indicated that high concentrations of exosomes (50–150 nm) are contained within HuNoV-infected stool. Extracellular vesicle (EV).


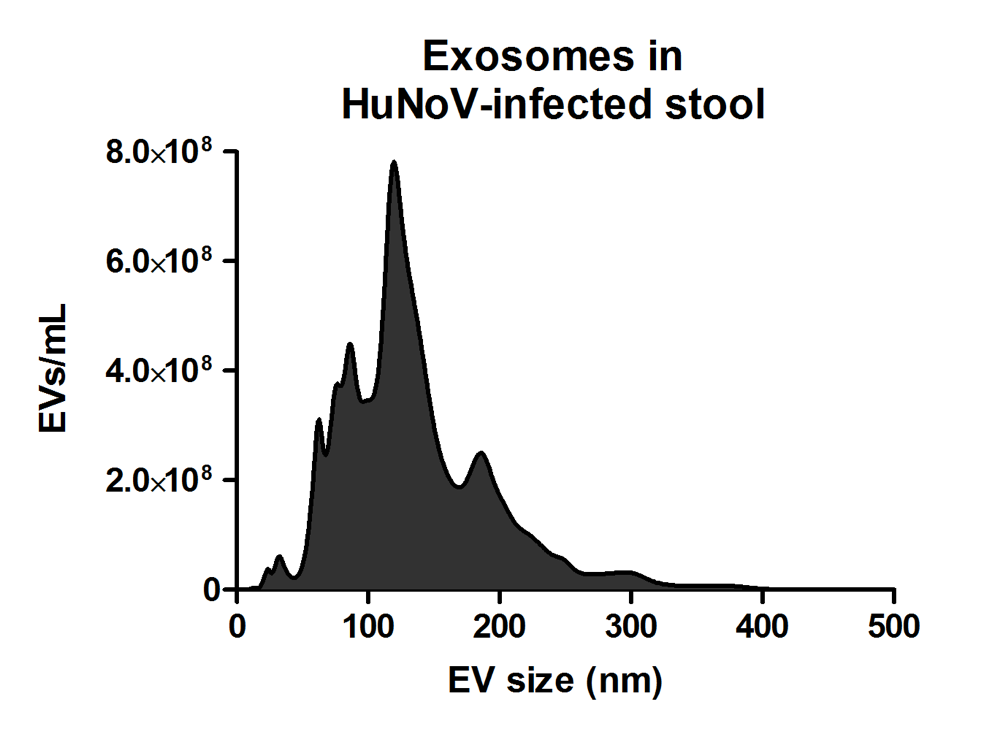

Supplement: S1 Fig — NanoSight analysis indicated that high concentrations of exosomes (50–150 nm) are contained within HuNoV-infected stool. Extracellular vesicle (EV). (DOCX) [file pone.0237044.s001.docx]
