# Supplementary figures and images for: Exosome-mediated human norovirus infection
Source: PLoS One. 2020 Aug 3;15(8):e0237044. doi: 10.1371/journal.pone.0237044 (PMC7398508; doi:10.1371/journal.pone.0237044)

**S2 Fig. Sample HuNoV g.e. standard curve.**

**
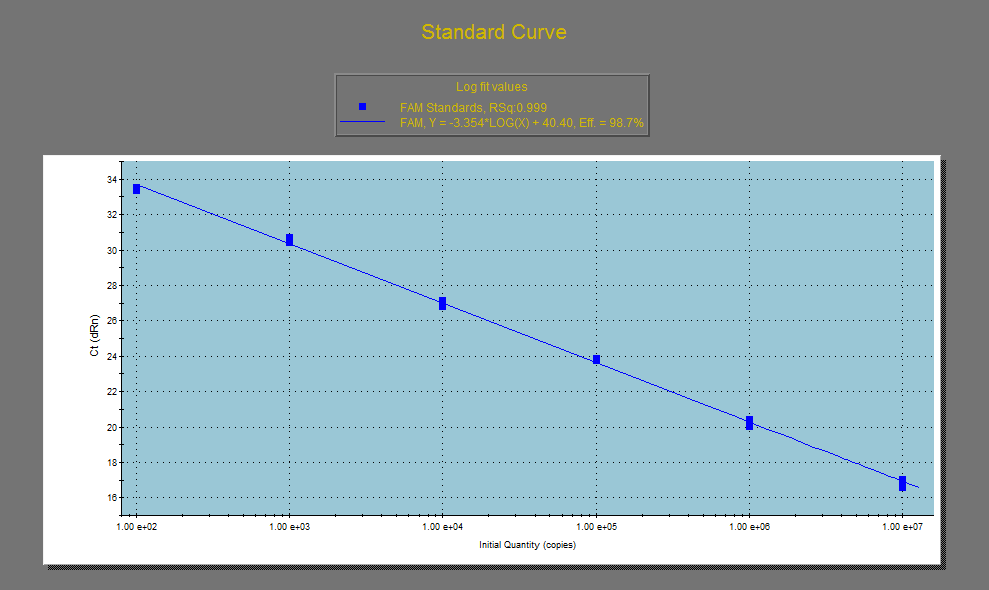
**

Supplement: S2 Fig — (DOCX) [file pone.0237044.s002.docx]
