# Supplementary material for: Exosome-mediated human norovirus infection
Source: PLoS One. 2020 Aug 3;15(8):e0237044. doi: 10.1371/journal.pone.0237044 (PMC7398508; doi:10.1371/journal.pone.0237044)
Supplement: S1 Table — (DOCX) [file pone.0237044.s003.docx]

**S1 Table. Mean Ct values associated with Table 2.**

| **Virus** | **Sample** | **Time-point** | **Mean Ct ± SEM** |
| --- | --- | --- | --- |
| GII.4 Sydney | PS-exosomes | 0h | 34.46 ± 0.25 |
|  |  | 72h | 33.92 ± 0.24 |
|  | PS-depleted | 0h | 33.37 ± 0.21 |
|  |  | 72h | 32.41 ± 0.19 |
| GII.3 | PS-exosomes | 0h | 33.15 ± 0.21 |
|  |  | 72h | 32.70 ± 0.33 |
|  | PS-depleted | 0h | 33.17 ± 0.18 |
|  |  | 72h | 31.49 ± 0.36 |
| GII.4 Den Haag | stool | 0h | 34.70 ± 0.34 |
|  |  | 72h | 34.37 ± 0.27 |
|  | PS-exosomes | 0h | 34.61 ± 0.31 |
|  |  | 72h | 34.18 ± 0.13 |
|  | PS-depleted | 0h | 34.48 ± 0.18 |
|  |  | 72h | 34.88 ± 0.23 |

Abbreviations: threshold cycle (Ct) and standard error of the mean (SEM).
