# Supplementary material for: Exosome-mediated human norovirus infection
Source: PLoS One. 2020 Aug 3;15(8):e0237044. doi: 10.1371/journal.pone.0237044 (PMC7398508; doi:10.1371/journal.pone.0237044)
Supplement: S2 Table — (DOCX) [file pone.0237044.s004.docx]

**S2 Table. Mean Ct values associated with Table 3.**

| **Virus** | **Sample** | **Time-point** | **Mean Ct ± SEM** |
| --- | --- | --- | --- |
| GII.4 Sydney | stool | 0h | 33.30 ± 0.63 |
|  |  | 72h | 32.80 ± 0.67 |
|  | exosomes | 0h | 31.92 ± 0.58 |
|  |  | 72h | 31.43 ± 0.57 |

Abbreviations: threshold cycle (Ct) and standard error of the mean (SEM).
